# Aging gene signature of IL-7 receptor alpha low effector memory CD8^+^ T cells is associated with neurocognitive functioning in Alzheimer’s disease

**DOI:** 10.21203/rs.3.rs-2736771/v1

**Published:** 2023-04-04

**Authors:** Juan Young, Hong-Jai Park, Minhyung Kim, Jennefer Par-Young, Hugh Bartlett, Hye Sun Kim, Serhan Unlu, Lais Osmani, Min Sun Shin, Richard Bucala, Christopher van Dyck, Heather Allore, Adam Mecca, Sungyong You, Insoo Kang

**Affiliations:** Yale School of Medicine; Yale School of Medicine; Cedars-Sinai Medical Center; Yale University; Rutgers Robert Wood Johnson Medical School; Yale School of Public Health; Cleveland Clinic Fairview Hospital; Yale School of Medicine; Yale University; Yale University; Yale School of Medicine; Yale; Yale University School of Medicine; Cedars-Sinai Medical Center; Yale School of Medicine

**Keywords:** Aging, Alzheimer’s disease, senescence, T cell, adaptive immunity, transcriptomics

## Abstract

CD45RA^+^ effector memory (EM) CD8^+^ T cell expansion was reported in Alzheimer’s disease (AD). Such cells are IL-7 receptor alpha (IL-7Rα)^low^ EM CD8^+^ T cells, which expand with age and have a unique aging gene signature (i.e., IL-7Rα^low^ aging genes). Here we investigated whether IL-7Rα^low^ aging genes and previously reported AD and memory (ADM) genes overlapped with clinical significance in AD patients. RT-qPCR analysis of 40 genes, including 29 ADM, 9 top IL-7Ra^low^ aging and 2 control genes, showed 8 differentially expressed genes between AD and cognitively normal groups; five (62.5%) of which were top IL-7Rα^low^ aging genes. Over-representation analysis revealed that these genes were highly present in molecular and biological pathways associated with AD. Distinct expression levels of these genes were associated with neuropsychological testing performance in 3 subgroups of dementia participants. Our findings support the possible implication of the IL-7Rα^low^ aging gene signature with AD.

## Introduction

Alzheimer’s disease (AD) is a chronic progressive neurodegenerative disorder and the most common cause of dementia, accounting for approximately 60–80% of all dementia cases (1). Amyloid-β (Aβ), a peptide derived from the amyloid precursor protein (APP) and known to assemble into extracellular amyloid plaques, is hypothesized to initiate AD pathogenesis by stimulating the formation of neurofibrillary tangles and contributing to the development of a cytotoxic environment prone to neurodegeneration (2). Successive stressful events early in life including infections, ischemia, free radicals, and metabolic insults are thought to result in a chronic, low-intensity in ammatory state that stimulates pathological Aβ accumulation and the development of the AD dementia syndrome (3, 4). Previous studies have focused primarily on the innate immune system, including microglial cells which can be activated by Aβ, as a direct approach to evaluating neuroin ammation in the central nervous system (CNS) (5). In contrast, fewer have investigated the potential role of adaptive immunity, including T cells, on AD pathology as part of a dysregulated systemic immune response resulting from harmful interactions between CNS immune cells and the peripheral immune system (6, 7).

Aging is the strongest risk factor for AD (1). In human T cells, probably the most prominent change with aging is the expansion of effector memory (CCR7^−^,EM) CD8^+^ T cells, which include CD45RA^+^ and CD45RA^–^ populations (**note:** hereafter EM indicates both CD45RA^+^ and ^−^ populations unless specified), in peripheral blood (8, 9). One potential contributor to AD pathology includes cytotoxic and senescent T cell populations that may interact with the CNS through disruptions in the blood brain barrier (7, 10). Previously, our lab found an age-associated expansion of peripheral in ammatory and cytotoxic human effector memory (EM, CCR7^−^CD45RA^+/−^) CD8^+^ T cells expressing low levels of IL-7 receptor alpha chain (IL-7Rα or CD127)^low^, which have distinct characteristics including effector molecules, transcription factors, and DNA methylation profiles (10–13). Such T cell expansion, which is likely driven in part by repetitive immune stimulation over a lifetime (14), contributes to age-associated transcriptomic changes in human peripheral blood cells. About 15% of the age - associated genes (231/1,497) reported by a meta analysis study of human peripheral blood from approximately 15,000 individuals corresponded to differentially expressed genes (DEGs) in IL-7Rα^low^ EM CD8^+^ T cells (15).

A recent study reported the expansion of CD45RA^+^ EM CD8^+^ T cells (T_EMRA_) in the cerebrospinal fluid of patients with dementia or mild cognitive impairment (MCI) due to AD (16). The clonal expansion of these cells in peripheral blood was found to also correlate with cognitive scores. Importantly, such cells are mostly IL-7Rα^low^ EM CD8^+^ T cells, implying the possible relationship of such cell expansion with transcriptomic alterations in the peripheral blood of individuals with AD. Thus, the present study was conducted to determine if an altered IL-7Rα^low^ EM CD8^+^ T cell associated aging gene signature (i.e., IL-7Rα^low^ aging genes) occurs in peripheral blood of participants with AD, especially in relation to genes identified to be possibly associated with AD by our systemic selection process of publicly available datasets, compared to cognitively normal older adults (CN).

## Results

### A systemic search of publicly available data reveals a set of AD and memory-associated genes that are also found in the IL-7Rα^low^ aging gene signature.

**Figure 1:**
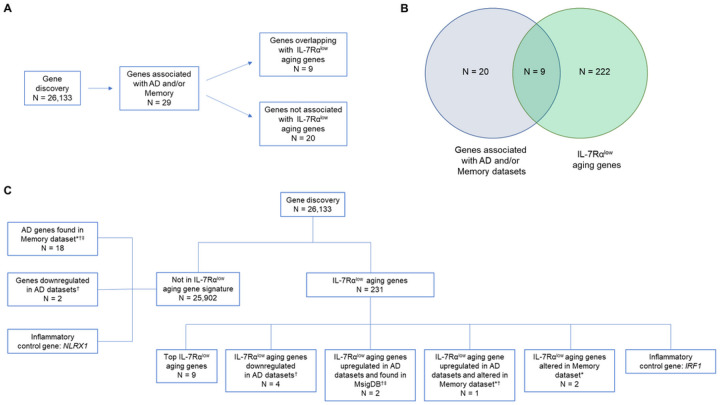
Gene discovery diagrams (A-B) Gene discovery involved the analysis of 26,133 total genes based on publicly available databases that included AD (GSE140829, and GSE63060 and GSE63061) and memory (GSE127711) transcriptomic datasets as well as the MSigDB. This resulted in the discovery of 29 genes associated with AD and/or memory. Of the 29 genes, 9 were IL-7 receptor alpha low (IL-7Rα^low^) aging genes (n = 231). (C) Gene discovery diagram showing the distinct characteristics of the analyzed gene categories. In addition to the genes identified by a systemic search of publicly available data, 2 inflammatory control genes and the top IL-7Rα^low^ aging genes (i.e., IL-7Rα^low^ aging genes most associated with chronological age) were also studied. Molecular Signatures Database (MSigDB), *Altered in memory dataset, ^†^Differentially expressed in 2 or more AD datasets, ^‡^Found in MSigDB

We explored whether any IL-7Rα^low^ aging genes (n = 231), which we reported in our published study (15), also belonged to genes possibly associated with AD. To select the latter gene set possibly associated with AD, we performed a systematic gene selection process on a large set of transcriptomic data derived from public domain gene set databases. We identified 3 publicly available peripheral blood transcriptomic datasets (GSE140829, GSE63060 and GSE63061) from a combined total of 428 patients with AD and 554 cognitively normal subjects and selected a set of genes which were differentially expressed in ≥ 2 of these datasets (17, 18). We also utilized a blood-based biomarker “memory” dataset (GSE127711) to identify genes of interest associated with short term memory dysfunction. The memory dataset was generated from a study which carried out longitudinal within-subject analysis in male and female psychiatric patients to identify blood gene expression biomarkers that tracked short term memory as measured by the retention measure in the Hopkins Verbal Learning Test (19). In addition, by searching the Molecular Signatures Database (MSigDB) (20) with keywords “Alzheimer” and “Alzheimer’s”, we identified the following gene sets: KEGG_ALZHEIMERS_DISEASE (n = 166 genes), BIORCARTA P35ALZHEIMERS_PATHWAY (n = 8 genes), BLALOCK_ALZHEIMERS_DISEASE_DN (n = 1248 genes down-regulated in brains from patients with AD) (21), BLALOCK_ALZHEIMERS_DISEASE_UP (n = 1671 genes up-regulated in brains from patients with AD) (21), WU_ALZHEIMER_DISEASE_DN (n = 19 genes down-regulated in brain endothelial cells from patients with AD) (22), WU_ALZHEIMER_DISEASE_UP (n = 14 genes up-regulated in brain endothelial cells from patients with AD) (22), and RAY_ALZHEIMERS_DISEASE (n = 13 genes from a biomarker study of plasma signaling proteins that predicted clinical Alzheimer’s diagnosis) (23). This systemic data search revealed 29 AD and/or memory-associated genes of which 9 genes (31%) were IL-7Ra^low^ aging genes (P < 0.001) (Figures 1A–C).

### IL-7Rα^low^ aging genes are differentially expressed in the peripheral blood of patients with AD as determined by RT-qPCR.

**Figure 2:**
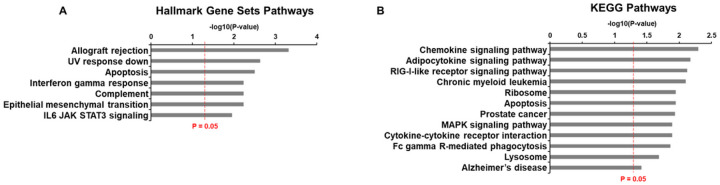
Hallmark gene sets and KEGG pathways enriched by analyzed genes. Hallmark gene sets (A) and KEGG (B) pathways identi ed by gene set enrichment analysis (GSEA) of the 40 analyzed genes.

We next investigated whether the AD-associated genes identified through our systematic approach, including ones belonging to the IL-7Rα^low^ aging gene signature, were altered in an independent cohort from the Yale ADRC. We also included 9 genes which were previously identified by our group to be differentially expressed in IL-7Rα^low^ EM CD8^+^ T cells and the most highly associated with chronological aging as we previously reported (referred to as top IL-7Rα^low^ aging genes), and two inflammatory control genes (IRF1 and NLRX1) from IL-7Rα and non-IL-7Rα, respectively, which were not found in the AD or memory datasets though they have the potential to affect AD pathology (15, 24). These genes (n = 40) were found to be enriched in IL-6 signaling, interferon gamma response, complement, chemokine signaling, and cytokine-cytokine receptor interaction pathways in addition to AD (Figures 2A–B). Indeed, these pathways have been suggested to be associated with AD though these genes have not yet been validated through more specific modalities of transcriptomic analyses such as RT-qPCR (25–28). See Supplementary Table S2 for a list of the analyzed genes and Supplementary Methods 1.2 for more information.

**Figure 3:**
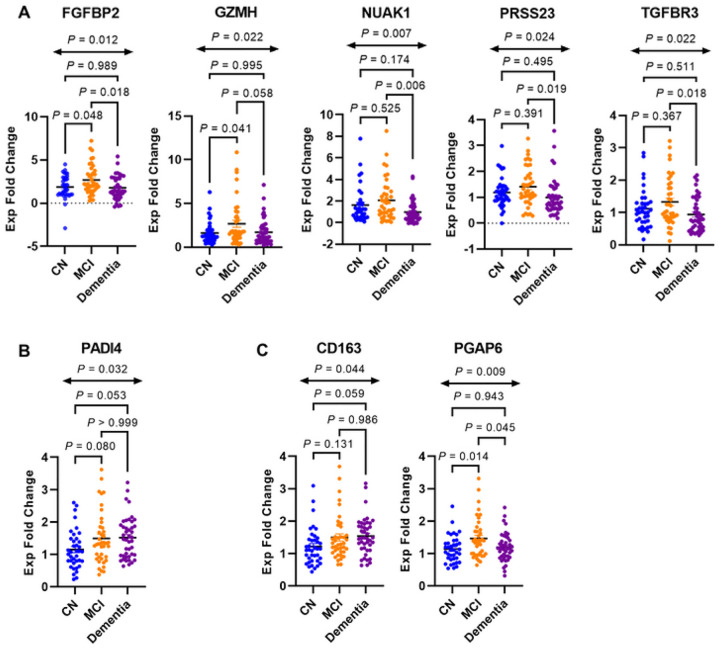
A set of genes associated with IL-7 receptor alpha (low) effector memory CD8^+^ T cells and Alzheimer’s disease (AD) is differentially expressed in peripheral blood of patients with AD. Reverse transcription qPCR (RT-qPCR) analysis showing differentially expressed genes in peripheral blood of cognitively normal (CN) and AD participants with mild cognitive impairment (MCI) or dementia. (A) Top IL-7 receptor alpha low (IL-7Rα^low^) effector memory (EM) CD8^+^ T cell-associated aging genes. (B) PADI4, an IL-7Rα^low^ aging gene found to be upregulated in 3 AD transcriptomic datasets. (C) Genes associated with MSigDB, memory and AD transcriptomic datasets but not in the IL-7Rα^low^ EM CD8^+^ T cell associated aging gene signature. P values were obtained by ANOVA and adjusted during post-hoc multiple comparison testing using the Šidák correction.

Eight genes (20%) out of the 40 analyzed were found to be differentially expressed in the peripheral blood of the CN, MCI, and dementia groups (Figure 3, see Supplementary Figures S1.1-S1.7. for all other gene expression plots). Six of the eight genes were IL-7Rα^low^ aging genes (75%, P-value = 0.120) (15), including FGFBP2, GZMH, NUAK1, PRSS23, TGFBR3 and PADI4 (Figure 3A–B). Of these, five of the six genes were top IL-7Rα^low^ aging genes (62.5%, P-value = 0.002) (Figure 3A) (15). The gene encoding for *f*ibroblast growth factor binding protein 2 (FGFBP2) was found to be more highly expressed in the MCI group compared to the CN group (P-value = 0.048) and dementia group (P-value = 0.018). GZMH, a gene which encodes for the human serine protease granzyme H, was more highly expressed by the MCI group compared to the CN group (P-value = 0.041). NUAK1 (gene which encodes for a protein kinase), PRSS23 (gene that encodes for a serine protease of the trypsin family), and TGFBR3 (gene that encodes for the transforming growth factor (TGF)-β type III receptor) were found to have high levels of expression in the MCI group compared to the dementia group. These findings indicate increased expression of four of the top IL-7Rα^low^ aging genes in individuals with MCI as compared to those with dementia. Of the genes associated with the IL-7Rα^low^ EM CD8^+^ T cells and AD (based on the 3 publicly available AD datasets evaluated), PADI4, which encodes peptidyl arginine deiminase 4 and is related to chromatin organization and protein citrullination by converting arginine residues to citrulline residues, was found to be differentially expressed among the three clinical groups though adjusted P-values from post-hoc multiple comparison testing did not reach statistically significant levels of differences in gene expression between clinical groups (Figure 3B).

Two genes associated with AD (per both MSigDB and AD microarray datasets) (17, 18, 20) and the memory dataset (19) (but were not IL-7Rα^low^ aging genes) were found to be differentially expressed (Figure 3C). CD163 encodes scavenger receptor cysteine-rich type 1 protein M130 expressed by monocytes and macrophages. This gene was found to be differentially expressed among the three clinical groups, though adjusted P-values generated from post-hoc multiple comparison testing suggest no statistically significant differences between clinical groups. PGAP6, which encodes post-glycosylphosphatidylinositol attachment to proteins 6, was also found to be differentially expressed with higher expression levels in the MCI group compared to both the CN (P-value = 0.014) and dementia (P-value = 0.045) groups.

We performed GLM analysis to adjust for age, race, and sex. A full-factorial model with all possible variables, covariates, and interactions (i.e., age, sex, race, diagnosis and all interaction terms) was generated, and predictors that were not significant were removed from the predictive model. Four genes (FGFBP2, PRSS23, TGFBR3, NUAK1) out of 5 top IL-7Rα^low^ aging genes remained differentially expressed at the P < 0.05 significance level after Šidák correction with decreased relative gene expression in the dementia group compared to the MCI group (Supplementary Tables S3–4). Further analysisof the genes differentially expressed in the public AD or memory datasets identified PGAP6, which is up-regulated in the MCI group compared to the dementia group (adjusted P-value = 0.029), and AKAP13 (encodes A-kinase anchoring protein 13) which was differentially expressed between the CN and dementia groups (adjusted P-value = 0.023).

### Over-representation analysis (ORA) of DEGs demonstrates molecular and biological pathways associated with AD.

**Figure 4:**
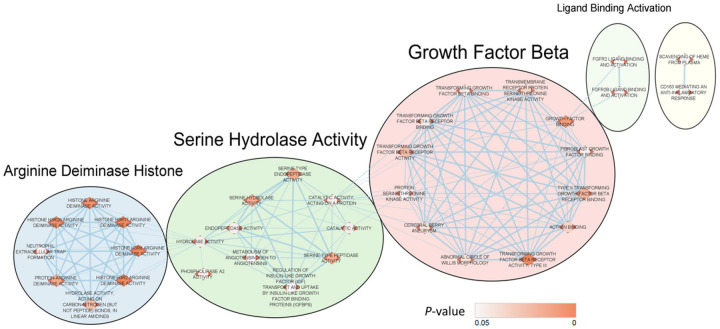
Differentially expressed genes (DEGs) are over-represented in pathways associated with Alzheimer’s disease (AD). Over-representation analysis showing the 8 DEGs in Fig 3 were over-represented in molecular and biological pathways thought to be linked to AD including TGF-β signaling, serine hydrolase activity, and histone modi cation. Auto-annotation descriptions of the 3 major clusters displayed corresponded with the pathways that were most significantly over-represented by the DEGs.

To determine if the 8 DEGs discovered through qPCR were also over-represented or more present in known biological and molecular interactions/pathways, ORA was conducted utilizing the online program g:Profiler with the Benjamini-Hochberg FDR multiple testing correction method applying a significance threshold of 0.05 (29). Over-representation of the DEGs in these pathways was compared to a background list of genes (see Supplementary File Background Gene List). The pathways analyzed were obtained from readily available online databases including Gene Ontology (GO) pathways describing molecular function, cellular components, and biological processes; biological pathway databases including KEGG, Reactome, and WikiPathways; and the Human Phenotype Ontology database. The analysis identified multiple pathways that the 8 DEGs were highly over-represented. Such pathways included those annotated to describe histone modification with arginine deiminase activity, serine-type hydrolase activity, and TGF-β signaling (Table S5); all of which were previously associated with AD (30, 31) or have been suggested as potential pharmacologic targets (32). To aid with interpretation of this analysis, Cytoscape applications were utilized to produce a network map (Figure 4) that organizes and automatically generates networks of overlapping gene sets that infer interactions between the identified pathways (33, 34). A word-based clustering algorithm was also utilized to group these pathways based on the highest similarity in 3-word descriptions found in the general description annotations of highly enriched biological pathways and gene ontology terms uncovered in the ORA (33, 35, 36). Complementing the ORA findings listed in Table S5, the pathways most significantly over-represented by the DEGs paralleled the 3 most common word descriptions (which were generated without supervision) annotating each pathway cluster as indicated by the annotations “Growth Factor Beta”, “Serine Hydrolase Activity”, and “Arginine Deiminase Histone” (Figure 4). Additionally, edges (i.e., lines connecting nodes denoting associations between enriched pathways) generated between the 3 major clusters in the enrichment map imply potential functional, molecular, and biological interactions that could become robust targets of investigation for future studies of AD pathology.

### Neurocognitive function is associated with IL-7Rα^low^ aging gene expression in dementia due to AD.

To uncover potential associations between aging genes and AD status, principal component analysis (PCA) was performed using Z-scores calculated from the expression levels of all 40 genes of interest (Supplementary Figure S2). Minimal separation or clustering based on disease status was observed. Unbiased hierarchal clustering (Supplementary Figure S3) was also performed to reveal trends of gene expression in different gene target groups which demonstrated substantial portions of the MCI group and a small part of the dementia group clustered together with high expression levels of the top aging genes associated with IL-7Rα ^low^ EM CD8^+^ T cells (represented by the “Top IL-7Rα^low^ aging genes” category in Supplementary Figure S3, the cluster indicated by a blue arrow), though there was more variability and heterogeneity in clustering of the rest of the participants.

**Figure 5:**
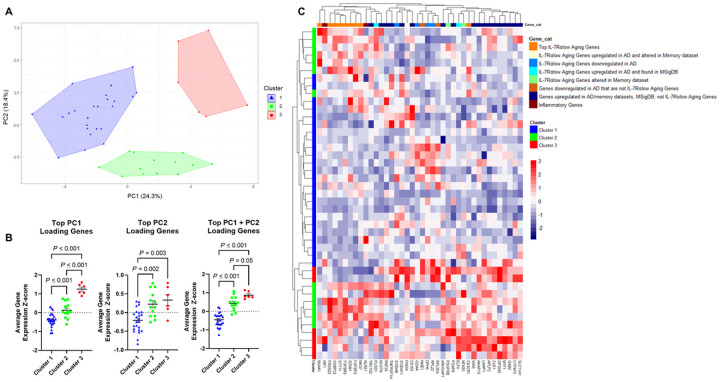
Unbiased clustering analyses of gene expression in the dementia group reveal three clusters of dementia patients with distinct levels of IL-7 receptor alpha (low) aging gene expression. (A) Principal component analysis (PCA) was done based on the expression levels of the 40 analyzed genes in peripheral blood of dementia participants. PCA yielded three clusters: Cluster 1 (blue triangles), Cluster 2 (green boxes), and Cluster 3 (red circles). (B) Average gene expression Z-scores of the top 10 loading genes for each principal component (PC) in the 3 clusters identi ed from (A) by PCA analysis of the dementia group. (C) Heatmap showing the results of unbiased hierarchal clustering analysis of gene expression in dementia participants. Subject clusters are based on PCA analysis in (A). P values were obtained by ANOVA with post-hoc multiple comparison testing or Welch’s t-test.

Subgroup analysis of the dementia group was completed due to notable clustering of dementia participants according to PC1 and PC2 based on the PCA analysis of all participants (Supplementary Figure S2). PCA analysis of the dementia group was conducted and revealed three distinct clusters within this group (Figure 5A). Notably, Cluster 1 dementia participants had lower average gene expression Z-scores of PC1 + PC2 top loading genes compared to both Clusters 2 and 3, suggesting similar gene expression patterns between the latter two clusters (Figure 5B). Supporting this, unbiased hierarchal clustering (Figure 5C) demonstrated a relative increase in gene expression levels of the top IL-7Rα^low^ aging genes in Clusters 2 and 3 compared to Cluster 1.

**Figure 6:**
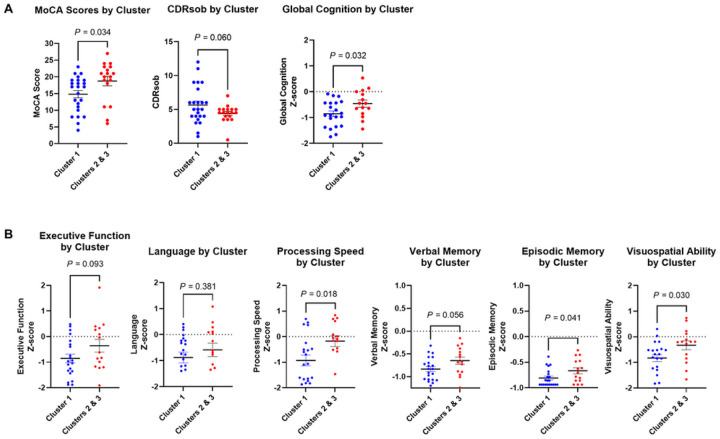
Clusters in dementia group associated with higher IL-7 receptor alpha (low) aging gene expression exhibit higher cognitive functioning according to comprehensive neuropsychological testing. (A) MoCA scores, CDRsob scores, and Global Cognition composite scores of Cluster 1 compared to Clusters 2 & 3 combined. Clusters 1, 2 and 3 were identified based on PCA analysis in Figure 5A. (B) Neuropsychological testing Z-scores of individual cognitive domains plotted according to dementia clusters. *P*values were obtained by unpaired t-test. MoCA, Montreal Cognitive Assessment; CDRsob, Clinical Dementia Rating scale sum of boxes.

**Figure 7:**
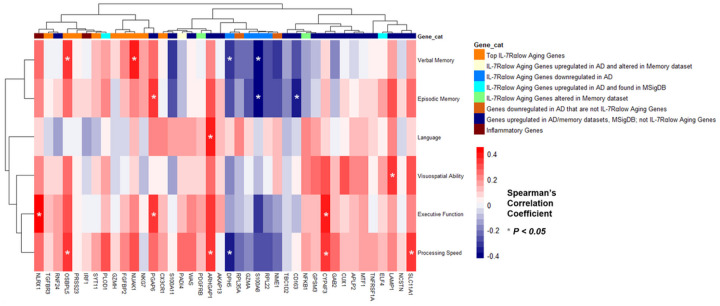
Top IL-7 receptor alpha (low) aging gene expression levels significantly correlate with neuropsychological testing scores in over half of the tested cognitive domains in participants with dementia. Unbiased hierarchal clustering heatmap of dementia participants’ Spearman’s rho coefficients (reflecting correlations between neuropsychological testing scores and all analyzed gene expression levels) were impartially categorized according to cognitive domain.

To evaluate neurocognitive functioning based on the 3 clusters of participants in the dementia group, MoCA and CDRsob scores (Supplementary Figure S4) as well as neuropsychological testing Z-scores (Supplementary Figure S5.1–5.6) were analyzed according to cluster designation. As Clusters 2 and 3 exhibited relatively similar gene expression profiles (Figure 5B–C) and similar MoCA and CDRsob scores (Supplementary Figure S4), both clusters were combined into one cluster which demonstrated significantly different MoCA scores (P-value = 0.034, Figure 6A) and Global Cognition Z-scores (P-value = 0.032, Figure 6A) compared to participants in Cluster 1. Furthermore, in comparing neuropsychological testing scores that re ect participants’ performance in several cognitive domains (i.e., episodic and verbal memory, executive function, processing speed, language, and visuospatial ability (37)), Cluster 1 participants had significantly lower neuropsychological testing scores compared to Clusters 2 and 3 in the domains of visuospatial ability, episodic memory, and processing speed (Figure B). Additionally, unbiased hierarchal clustering of Spearman’s rho coefficients reflecting correlation of gene expression levels with neuropsychological testing scores revealed clustering of top IL-7Rα^low^ aging genes based on their correlation with individual cognitive domain scores (Figure 7). Of the cognitive domains investigated, executive function, processing speed, and verbal and episodic memory scores were found to most correlate with expression of all genes analyzed (Figure 7). Of the top IL-7Rα^low^ aging genes, OSBPL5, a gene associated with proteins that function in lipid and cholesterol transport (38), was found to be significantly correlated with processing speed and verbal memory scores (Supplementary Figures S5.1–5.2) while NUAK1 was significantly correlated with verbal memory scores (Supplementary Figure S5.2).

## Discussion

AD is a disabling disease that is heterogeneous and multifactorial in its etiologies. This limits any one AD model from being globally representative of disease pathogenesis and the mechanisms that underly it (39). This difficulty in fully characterizing AD pathology drives the ongoing exploration into several potential pathways that may contribute to the development of AD. Studies of in ammatory pathways and their association with AD have been mounting in the past decade. Evidence of systemic immune dysregulation in AD is growing, though there is still a relative paucity of studies investigating the role of adaptive immunity, including T cells, in AD (40). Addressing the latter point is critical in that one of the most prominent changes with human aging is expansion of memory CD8^+^ T cells with senescent characteristics in peripheral blood (9). As AD incidence rates increase with age (annual incidence of AD appreciably increase above 75 years of age (41, 42)), it is possible that senescent T cells may contribute to AD progression through altered immune activity. This study aimed to expand upon contemporary notions of adaptive immune functioning in AD by investigating the potential presence and in uence of these senescent CD8^+^ T cells on AD through transcriptomic analysis and associating gene expression data with direct correlates of cognitive functioning. In addition, our study was conducted to validate publicly available microarray AD datasets generated from human peripheral blood with more specific transcriptomic analysis through RT-qPCR. The results of our study show the presence of an altered CD8^+^ T cell age-associated gene signature in the peripheral blood of patients with AD, warranting further studies investigating biological implications of CD8^+^ T cells, especially highly cytotoxic and in ammatory IL-7Rα^low^ EM CD8^+^ T cells, in AD.

Our systemic analysis of publicly available data revealed 29 AD and/or memory-associated genes of which 9 genes (31%) were aging genes enriched in IL-7Ra^low^ EM CD8^+^ T cells, suggesting the possible relationship of this cell subset with AD. The results of RT-qPCR analysis showed 8 DEGs between AD and CN groups. Of note, six (75%) of the 8 genes were IL-7Rα^low^ aging genes; five of which were top IL-7Rα^low^ aging genes. These “top IL-7Rα^low^ aging genes” have been previously associated with IL 7Rα^low^ EM CD8^+^ T cells (15), a T cell population characterized by expressing senescent markers that expand ‐ with age. These top IL-7Rα^low^ aging genes include genes related to cytotoxic molecules (FGFBP2, GZMH, PRSS23) as well pathways that have been associated with cell survival and cell cycle regulation (PRSS23, NUAK1) (43, 44). After adjusting for race, sex, and age using GLM analysis, four of nine (44%) of these top IL 7Rα^low^ aging genes (FGFBP2, NUAK1, PRSS23, TGFBR3) remained differentially expressed at a significance level of 0.05. Unsurprisingly, these genes have been previously associated with tumor growth and metastasis (NUAK1, TGFBR3, FGFBP2) (45, 46), as well as autoimmune disorders (PRSS23) (47). In relation to AD, NUAK1 overexpression is suspected to promote tau hyperphosphorylation based on findings in AD mouse models (48). Interestingly, these genes appeared more highly expressed in the MCI group compared to the CN and dementia groups. Similar patterns have been observed in a previous study which found increases in myeloid-derived suppressor cells and FOXP3^+^ CD4^+^ regulatory T cells in the peripheral blood of MCI participants compared to age-matched healthy and mild dementia participants (49). This pattern could be reflective of increasing immune activity and an initial anti-in ammatory response early in the disease course before the shift to full suppression of in ammation found in neurodegeneration.

ORA revealed that the 8 DEGs validated by our qPCR analysis were also highly present (over-represented) in known biological and molecular pathways previously associated with AD. Importantly, this analysis was able to demonstrate potential associations between different pathways that could lead to more robust investigations which could delineate relationships between molecular and biological processes that contribute to AD pathology. The most over-represented pathways have been individually associated with (or postulated to contribute to) AD in several studies. For example, peptidyl arginine deiminase was previously found to co-localize with amyloid beta-42 in the hippocampus and is postulated to mediate enhancement of histone modification and citrullination, thus promoting neutrophil extracellular trap formation which would contribute to the pro-inflammatory milieu in AD (50, 51). Inhibition of acetylcholinesterase, a metabolic serine hydrolase, has been a mainstay of treating cognitive symptoms in patients with AD (32). Lastly, dysfunctional TGF-β signaling has been reported as a possible cause of amyloid beta accumulation and neurodegeneration (52). Thus, this network-based analysis provides a framework in investigating the relationship between these three major pathways which could lead to development of potential biomarkers or therapeutic targets.

Regarding associating differential expression with the clinical phenotypes of AD, subgroup analyses of the clinical groups revealed distinct clustering and separation of dementia participants in PCA and unbiased hierarchal clustering. Dementia participants that were separated into Clusters 2 and 3 were noted to exhibit higher gene expression levels of IL ‐7Rα^low^ aging genes compared to Cluster 1 participants. Interestingly, Clusters 2 and 3 also exhibited higher neuropsychological testing scores suggesting a higher level of cognitive functioning in participants with higher relative T cell associated aging gene expression than in participants with a more progressed dementia syndrome. This may be reflective of the heterogeneity in AD disease progression with a subset of dementia participants that could also be experiencing altered immune functioning. A recent study reported the expansion of CD45RA^+^ EM CD8^+^ T cells in the cerebrospinal uid of patients with dementia or MCI due to AD, as well as the correlation of such cell expansion in peripheral blood with cognitive scores (16). CD45RA^+^ EM CD8^+^ T cells expanded in the CSF were IL-7Rα^low^. These findings support the results of our study revealing the possible relationship of the IL-7Rα^low^ aging signature with cognitive function in the dementia group.

There are several limitations of this study. Participants were matched according to chronological age to minimize confounding due to this variable. However, there are other potential confounding variables (e.g., biological aging) that we are unable to control for and could affect the potential homogeneity age-matching could produce across participants. Additionally, the low participation rates of under-represented peoples in the MCI and dementia groups could be a limitation of our study. Lastly, further studies are warranted if peripheral blood gene expression is reflective of immune alterations found in the CNS.

Altogether, the gene expression patterns in this AD cohort are suggestive of an altered immune response compared to healthy normal aging. Critically, there is signi cant differential gene expression of several aging genes associated with IL‐7Rα^low^ EM CD8^+^ T cells, and expression patterns of such genes could divide individuals with dementia due to AD into groups with different levels of cognitive functioning. Taken together, our findings allude to the potential impact of peripheral immune dysregulation in contributing to neuroin ammatory processes associated with AD pathology, warranting further investigations of these transcriptomic findings in AD.

## Online Methods

### Participant Characteristics

A total of 121 whole blood samples from participants recruited by the Yale Alzheimer’s Disease Research Center (ADRC) were requested and obtained for processing and transcriptomic analysis (Table 1). To estimate the minimum sample size needed to demonstrate differential gene expression between CN and AD participants at a signi cance level of 0.05, power calculations were done (with an assumed power of 0.8) utilizing preliminary gene expression data of the rst 60 participants included in this study. The Yale University Institutional Review Board approved the collection and processing of all whole blood samples investigated in this study. Informed consent was obtained from all participants at the time of collection by ADRC staff. Participants included in this study were organized into the following clinical groups based on consensus diagnosis: CN participants, MCI due to AD, and probable dementia due to AD. MCI and probable dementia due to AD are defined according to the National Institute on Aging – Alzheimer’s Association (NIA-AA) guidelines (53, 54). Clinical data from each participant, including biomarkers and cognitive testing scores, were utilized to produce a consensus diagnosis based on review by a multidisciplinary panel of experts from the Yale ADRC. Severity and staging were assessed using clinical rating scales including the Montreal Cognitive Assessment (MoCA) (55), and Washington University’s Clinical Dementia Rating (CDR) scale (56). Cognitive functioning was also evaluated utilizing the neuropsychological testing battery from the National Alzheimer’s Coordinating Center’s Uniform Data Set. The scores from this comprehensive cognitive testing were used to generate composite Z-scores for cognitive domains including episodic and verbal memory, executive function, processing speed, language, and visuospatial ability (37). These Z-scores were averaged for each participant to generate a “Global Cognition” Z-score to represent the overall cognitive functioning of each participant based on the neuropsychological testing results. Of the participants included in this study, 38 were CN, 40 were diagnosed with MCI due to AD, and 43 were diagnosed with dementia due to AD. Though the three clinical groups were age-matched and were similar in distribution in male and female participants, they were found to have an unequal distribution of participants based on the following racial categories: White, Black, American Indian/Native Alaskan, Asian, or Unknown (i.e., participants that declined to identify with any single race).

### RNA Isolation and Complementary DNA synthesis

RNA was isolated from peripheral whole blood stored in heparinized tubes at −80°C using a modi ed QIAGEN RNeasy Kit protocol (see Supplementary Methods 1.1). RNA templates were then utilized for complimentary DNA (cDNA) synthesis utilizing the iScript cDNA synthesis kit protocol (Bio-Rad).

### Quantitative Polymerase Chain Reaction

Target gene expression was measured by quantitative polymerase chain reaction (qPCR) analysis by producing 10 μl reaction mixtures containing cDNA, SYBR Green Supermix (Bio-Rad) and target gene primers at 1 μM concentrations (see Supplementary Table S1 for target gene primer RNA nucleotide sequences). One 384-well plate (Bio-Rad) was used for qPCR analysis of each analyzed gene to measure gene expression of all samples in one qPCR experiment in order to avoid experimental batch effects between clinical groups. β-actin was utilized as the housekeeping gene for all qPCR experiments. The reaction mixture was initially denatured at 95°C for 3 minutes and then underwent 40 cycles of the following: denatured for 15 seconds at 95°C then annealing, extension, and read uorescence for 45 seconds at 60°C using the CFX384 Touch Real-Time PCR Detection System (Bio-Rad). The expression levels for each gene were then calculated per the 2^−ΔΔC^_T_ equation (57).

### Data Processing

Approximately 2.6% of all expression fold change values comprising the current transcriptomic dataset were considered missing completely at random. Using Bioconductor’s “pcaMethods” software, missing values were imputed utilizing probabilistic principal component analysis (PPCA), a method of multiple imputation based on a probabilistic model created via a maximum likelihood estimation approach (58, 59). ComBat, a program in the Bioconductor’s “sva” software suite that utilizes empirical Bayes regression to adjust for and correct uncontrollable batch effects (60, 61) was used to process the transcriptomic dataset generated from the RT-qPCR analysis.

### Statistical Analyses

One-way ANOVA and Pearson’s chi-squared tests were performed as part of a descriptive analysis of ADRC participant demographic characteristics of age and sex, respectively. Due to the limited participation of non-White persons, analyses adjusting for race were conducted by labeling participants as either “non-Hispanic White” or “Other” with the latter group comprised of Hispanic and non-White participants. One-way ANOVA testing was completed to determine if there were differences in mean gene expression among the three clinical groups. General linear models (GLM) were also used to analyze differences in relative gene expression levels utilizing estimated least-square means (LSM) generated after adjusting for age, sex, and race. Fisher’s exact test was performed to calculate signi cance of overlap between 40 genes and gene sets. ORA was performed using g:Pro ler (version e108_eg55_p17_0254fbf) (29), and the following Cytoscape (version 3.9.1) (33) applications: EnrichmentMap version 3.35 (34), WordCloud version 3.1.4 (35), and AutoAnnotate version 1.4.0 (36). This was conducted using the Jaccard Overlap Combined Index test (k constant = 0.5) with an “Edge cutoff” of 0.62, P-value of 0.05, and q-value of 0.1. Welch’s t-test was conducted to determine if there were differences in MoCA scores, CDRsob scores, and Global Cognition Z-scores between dementia clusters. Unpaired t-tests were conducted to show any differences in neuropsychological testing cognitive domain Z-scores between dementia clusters. Spearman’s rho coefficients were calculated to determine if there were any signi cant correlations between neuropsychological testing scores and gene expression. All analyses were conducted to test for statistical signi cance in a “two-tailed” manner when applicable. Data was processed and analyzed using IBM SPSS Statistics for Windows, version 28.0, released in 2021, Armonk, NY: IBMCorp, R version 4.2.2, and GraphPad Prism version 9.5.0 for Windows, GraphPad Software, San Diego, California USA.

## Figures and Tables

**Table 1.** Demographics

	Cognitively Normal (CN) (N = 38)	Mild Cognitive Impairment (MCI) (N = 40)	Dementia (N = 43)	*P*-value
Age	72.3 ± 8.6	72.7 ± 7.4	71.2 ± 8.2	0.671
Female/Male	23 (60.5%) / 15 (39.5%)	17 (42.5%) / 23 (57.5%)	22 (51.2%) / 21 (48.8%)	0.282
Race				
Non-Hispanic White	22 (57.9%)	31 (77.5%)	40 (93.1%)	
Black	10 (26.3%)	4 (10%)	1 (2.3%)	
American Indian or Native Alaskan	2 (5.3%)	0	1 (2.3%)	0.009
Asian	1 (2.6%)	1 (2.5%)	0	
Unknown	3 (7.9%)	4 (10%)	1 (2.3%)	
Cognitive Scores				
MoCA	25.7 ± 3.2	20.3 ± 4.9	16.1 ± 6.3	<0.001
CDRsob	0.2 ± 0.5	2.2 ± 1.0	5.1 ± 2.3	<0.001

Demographic characteristics of 121 Alzheimer’s Disease Research Center (ADRC) participants involved in this study and organized according to clinical group. *P*-values were obtained by the ANOVA or Chi-square test. Abbreviations: MoCA. Montreal Cognitive Assessment: CDRsob. Clinical Dementia Rating sum of boxes

## Data Availability

This study included analysis of transcriptomic microarray data from publicly available datasets (GSE140829, GSE127711, GSE63060 and GSE63061) which are available from the Gene Expression Omnibus. Additional de-identi ed data utilized in this manuscript can be provided upon request from the Yale ADRC.
